# Impact of LTR-Retrotransposons on Genome Structure, Evolution, and Function in Curcurbitaceae Species

**DOI:** 10.3390/ijms231710158

**Published:** 2022-09-05

**Authors:** Shu-Fen Li, Hong-Bing She, Long-Long Yang, Li-Na Lan, Xin-Yu Zhang, Li-Ying Wang, Yu-Lan Zhang, Ning Li, Chuan-Liang Deng, Wei Qian, Wu-Jun Gao

**Affiliations:** 1 College of Life Sciences, Henan Normal University, Xinxiang 453007, China; 2 Institute of Vegetables and Flowers, Chinese Academy of Agricultural Sciences, Beijing 100081, China

**Keywords:** evolutionary dynamics, LTR-retrotransposons, Cucurbitaceae species, genome structure, gene expression

## Abstract

Long terminal repeat (LTR)-retrotransposons (LTR-RTs) comprise a major portion of many plant genomes and may exert a profound impact on genome structure, function, and evolution. Although many studies have focused on these elements in an individual species, their dynamics on a family level remains elusive. Here, we investigated the abundance, evolutionary dynamics, and impact on associated genes of LTR-RTs in 16 species in an economically important plant family, Cucurbitaceae. Results showed that full-length LTR-RT numbers and LTR-RT content varied greatly among different species, and they were highly correlated with genome size. Most of the full-length LTR-RTs were amplified after the speciation event, reflecting the ongoing rapid evolution of these genomes. LTR-RTs highly contributed to genome size variation via species-specific distinct proliferations. The Angela and Tekay lineages with a greater evolutionary age were amplified in *Trichosanthes anguina*, whereas a recent activity burst of Reina and another ancient round of Tekay activity burst were examined in *Sechium edule*. In addition, Tekay and Retand lineages belonging to the *Gypsy* superfamily underwent a recent burst in *Gynostemma pentaphyllum*. Detailed investigation of genes with intronic and promoter LTR-RT insertion showed diverse functions, but the term of metabolism was enriched in most species. Further gene expression analysis in *G.* *pentaphyllum* revealed that the LTR-RTs within introns suppress the corresponding gene expression, whereas the LTR-RTs within promoters exert a complex influence on the downstream gene expression, with the main function of promoting gene expression. This study provides novel insights into the organization, evolution, and function of LTR-RTs in Cucurbitaceae genomes.

## 1. Introduction

Long terminal repeat (LTR)-retrotransposons (LTR-RTs), one of the major groups of transposable elements (TEs) that can mobilize and replicate, are widespread in eukaryotic genomes [1]. They are particularly abundant in plants, making them major components of the plant genome. For example, LTR-RTs account for more than 75% and 70% of the nuclear genomes of maize and tea, respectively [2,3]. LTR-RTs are characterized by some typical structural features, such as the LTRs at each terminus and the adjacent target site duplications [4]. The internal region of LTR-RTs usually contains open reading frames for a GAG protein and a polymerase region (POL). POL encodes several enzymes crucial for the proliferation and integration of elements into the host genome, such as reverse transcriptase, RNA degradation enzyme RNaseH, and integrase [5]. According to their sequence similarity and the order of the reverse transcriptase and integrase coding regions, LTR-RTs are classified into two prominent superfamilies, Ty1-*copia* and Ty3-*gypsy* [6,7]. Each group can be further subdivided into a diversity of evolutionary lineages [6,8]. The main Ty1-*copia* lineages are Ale, Angela, Bianca, Ivana, TAR, Tork, and SIRE, while the most frequent Ty3-*gypsy* lineages are Athila, CRM, Galadriel, Ogre, Reina, Retand, and Tekay [8,9,10]. 

Similar to other retroelements, LTR-RTs transpose via an RNA intermediate using copy-and-paste transposition mode, which increases their copy number upon integration. Given their ability to proliferate and attain a very high copy number, LTR-RTs are often responsible for the expansion of the host genome. A number of studies demonstrated that LTR-RTs serve as a major driving force for genome size evolution [2,3,11,12,13]. In addition, studies of model and non-model species have shown that LTR-RTs exert tremendous effects on shaping chromosome structure [14], maintenance of genome stability [15], formation of specific genome regions [16,17], gene exonization and intronization [18,19], and gene regulation [20,21]. Specifically, several reports showed that LTR-RTs can influence the nearby gene expression and thereby change the phenotype of the species [22,23,24]. For example, an LTR-RT insertion upstream of the *MdMYB1* promoter is related to red-skinned fruit in apple [25]. Thus, comprehensive investigation of LTR-RTs is essential to understanding genome evolution and function. Recent efforts to characterize LTR-RTs in plant genomes add a new level of resolution to our understanding of the landscape and biological impact of these elements on genome evolution for individual organisms. However, few studies have focused on their dynamics on a family level. 

The botanical family Cucurbitaceae, also known as cucurbits and gourds, encompasses over 800 species that are distributed in nearly all arable regions worldwide [26]. This family is well known for its inclusion of various economically important cultivated plants, such as cucumber (*Cucumis sativus*), melon (*Cucumis melo*), and watermelon (*Citrullus lanatus*). At present, a number of Cucurbitaceae genomes have been sequenced and annotated [27,28,29,30]. Some of these genomes contain a large number of LTR-RTs and indicate LTR-RT proliferations within these species [31,32,33]. Thus, LTR-RTs may contribute to the structure and evolution of these Cucurbitaceae species. However, little attention has been paid to comparative studies of LTR-RTs among the Cucurbitaceae genomes to reveal their contributions to genome expansion and divergence. This study performed a systematic analysis of LTR-RTs in Cucurbitaceae genomes. The objectives of this study were (i) to establish the extent of intergeneric LTR-RT variations for both full-length LTR-RTs and LTR-RT fractions among different species, (ii) to study the relationship between LTR-RT abundance and genome size, (iii) to investigate variations in the LTR-RT dynamics among species, and (iv) to demonstrate the influence of LTR-RTs on the gene expression of related genes. 

## 2. Results

### 2.1. Phylogenetic Reconstruction of Cucurbit Species

**Figure 1:**
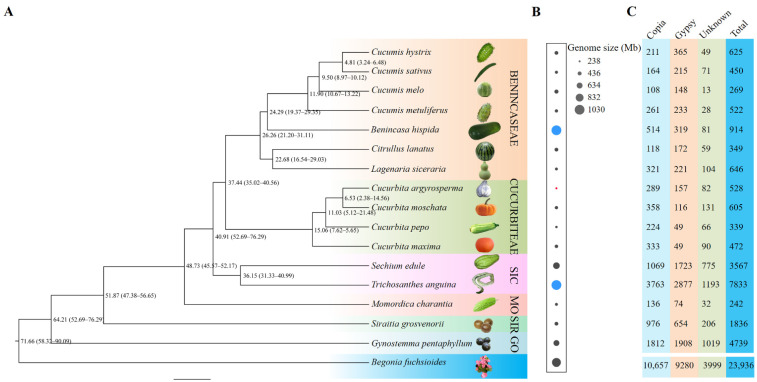
Phylogenetic analysis, genome size, and full-length LTR-RT numbers in Curcurbitaceae species. (**A**) Phylogenetic tree and divergence time. *Begonia fuchsioides* is used as an outgroup. Numbers on the nodes indicate the average divergence time of the common ancestor (MYA). The number ranges in the brackets represent the 95% confidence intervals of the estimated divergence time. Names of six tribes are noted at the right, and four of them are abbreviated: SIC, Sicyoeae; MO, Momordiceae; SIR, Siraitieae; GO, Gomphogyneae. (**B**) Genome size of the studied species. Red and blue dots indicate the smallest (*Cucurbita argyrosperma*, 238 Mb) and the largest genomes (*Benincasa hispida* and *Trichosanthes anguina*, 1030 Mb), respectively. (**C**) Number of full-length LTR-RTs detected in the 16 Curcurbitaceae species. The bottom row of numbers summarizes the total number of Copia, Gypsy, Unknown, and all LTR-RTs in the 16 Curcurbitaceae species.

In this work, 16 cucurbit species belonging to 10 genera representing six tribes were analyzed. Phylogenetic analysis and divergence time evaluation showed that the Curcurbitaceae family diverged from the common ancestor of the Begoniaceae family approximately 71.66 (58.32–90.09) million years ago (MYA). Over evolutionary time, the tribe Gomphogyneae including *Gynostemma pentaphyllum* and the tribe Siraitieae consisting of *Siraitia grosvenorii* diverged from the common ancestor approximately 64.21 (52.69–76.29) and 51.87 (47.38–56.65) MYA, respectively. The tribe Benincaseae including genera *Cucumis*, *Benincasa*, *Citrullus*, and *Lagenaria* formed a sister clade to the tribe Cucurbiteae. These two clades diverged approximately 37.44 (35.02–40.56) MYA. They were estimated to have diverged from the tribe Sicyoeae consisting of *Sechium* and *Trichosanthes* and the tribe Momordiceae including *Momordica charantia*, 48.73 (45.57–52.17) and 51.87 (47.38–56.65) MYA, respectively (Figure 1A). Among these species, the most recent speciation events occurred between the species *Cucumis hystrix* and *Cucumis sativus*; they diverged from the common ancestor about 4.81 (3.24–6.48) MYA (Figure 1A). The phylogenetic relationship and divergence time are highly consistent with previous studies [34]. 

The genome size of these cucurbit species showed a large variation. Overall, more than fourfold variation in genome size was detected, ranging from 238 Mb (*Cucurbita argyrosperma*) to 1030 Mb (*Benincasa hispida* and *Trichosanthes anguina*). The distribution of genome size along the phylogenetic tree is illustrated in Figure 1B, which showed distinct patterns of genome size evolution among different groups, with no general trend toward genome expansion or contraction. 

### 2.2. Identification and Annotation of Full-Length LTR-RTs 

A total of 23,936 full-length LTR-RTs were identified from the 16 cucurbit species, including 10,657 Ty1-*copia* (44.5%) and 9280 Ty3-*gypsy* elements (38.8%). However, 3999 LTR elements (16.7%) were not classified as *Copia* or *Gypsy* REs and, thus, designated as unknown elements. The size of the LTR-RTs ranged from 1173 bp to 28,350 bp, with a mean length of 6971 bp (standard deviation = 3321 bp). The terminal LTRs presented an average length of 996 bp with a standard deviation of 839 bp. 

**Figure 2:**
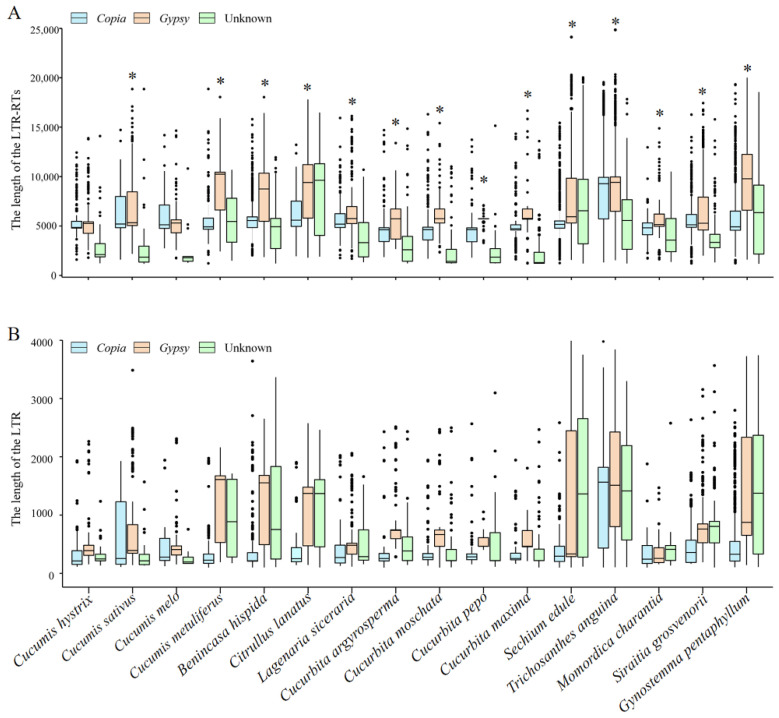
Length of the full-length LTR-RTs (**A**) and their LTRs (**B**) in Cucurbitaceae species. The asterisks (*) denote the 14 species with the average length of the *Gypsy* elements larger than that of the *Copia* and Unknown elements.

For each species, a remarkable variation across species in full-length LTR-RT number and cumulative length was discovered (Figure 1C). The number and cumulative length of LTR-RTs ranged from 242 (representing 1,230,197 bp in *M. charantia*) to 7833 (representing 63,807,282 bp in *T. anguina*). The densities (average number per Mb genome) also showed large variation. In general, small genomes were associated with low LTR-RT densities, such as 0.6 LTR-RTs/Mb in *C. melo* and 0.8 LTR-RTs/Mb in *M. charantia*, whereas large genomes showed high densities, such as 8.1 LTR-RTs/Mb in *G. pentaphyllum* and 8.5 LTR-RTs/Mb in *T. anguina*. The genome of *B. hispida* was a clear outlier, with an assembled genome size of 913 Mb and density of only 1 LTR-RTs/Mb (Table S1). In some species, the *Copia* elements were more than the *Gypsy* elements, such as in *B. hispida* and *Lagenaria siceraria*. However, in other species, the *Gypsy* elements were more abundant than *Copia* LTR-RTs, such as in *C. hystrix* and *C. sativus* (Table S1). The average length of LTR-RTs and the LTRs of *Copia*, *Gypsy*, and unknown elements within each species were calculated and compared. In general, the average LTR lengths were positively correlated with the average LTR-RT length. Interestingly, the average length of the *Gypsy* elements was strikingly larger than that of the *Copia* and unknown elements in the majority of species (14/16) (Figure 2 and Table S1). For example, in *G. pentaphyllum*, the average lengths of the *Gypsy* elements and their LTRs were 9505 and 1389 bp, respectively, whereas those of the *Copia*/unknown elements and corresponding LTRs were 5597/6141 and 509/1366, respectively. 

### 2.3. Genome Composition of LTR-RTs

**Figure 3:**
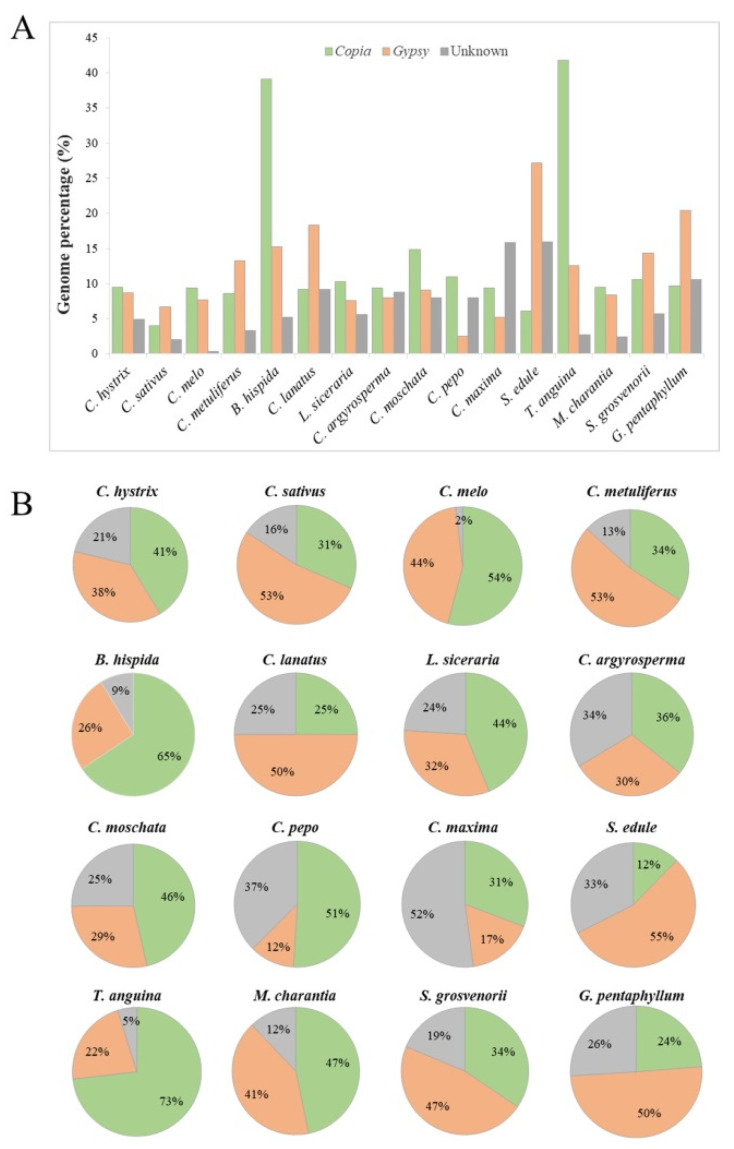
LTR-RT annotation of the genomes of 16 Cucurbitaceae species. (**A**) Genome proportion of *Copia*, *Gypsy*, and Unknown elements of each species. (**B**) Proportion of *Copia*, *Gypsy*, and Unknown elements in LTR-RT fractions in each species.

On the basis of the aforementioned full-length element, the genomic content masked by LTR-RTs ranged from 12.8% in *C. sativus* to 59.6% in *B. hispida* (Figure 3A). In accordance with the number of *Copia* and *Gypsy* LTR-RTs, the *Copia* and *Gypsy* contents varied among different species, with the ratio of *Copia* to *Gypsy* content ranging from 0.2 in *Sechium edule* to 4.4 in *C. pepo* (Figure 3B). It should be noted that only 914 full-length LTR-RTs were found in the genome of *B. hispida*, much less than that in *T. anguina* (7833), which had a similar genome size. However, the LTR-RT contents in these two genomes were similar. Correlation analysis was performed between genome size and full-length LTR-RT number/cumulative length or LTR-RT quantity/genome proportion in these studied Cucurbitaceae species. Results revealed a significant positive correlation between the genome size and the number of full-length LTR-RTs or the cumulative length (*R* = 0.7153 and 0.7541, respectively; *p* < 0.01, regression analysis) (Figure S1A,B). A much greater positive correlation was examined between the genome size and the total LTR-RT fraction, with *R* values of 0.9033 and 0.7769 for the total LTR-RT length and genome proportion, respectively (Figure S1C,D). These data suggest that the differential expansions of LTR-RTs greatly contributed to the upsize and downsize of the genomes among species. 

### 2.4. Evolutionary Dynamics of LTR-RTs in Cucurbitaceae Species

**Figure 4:**
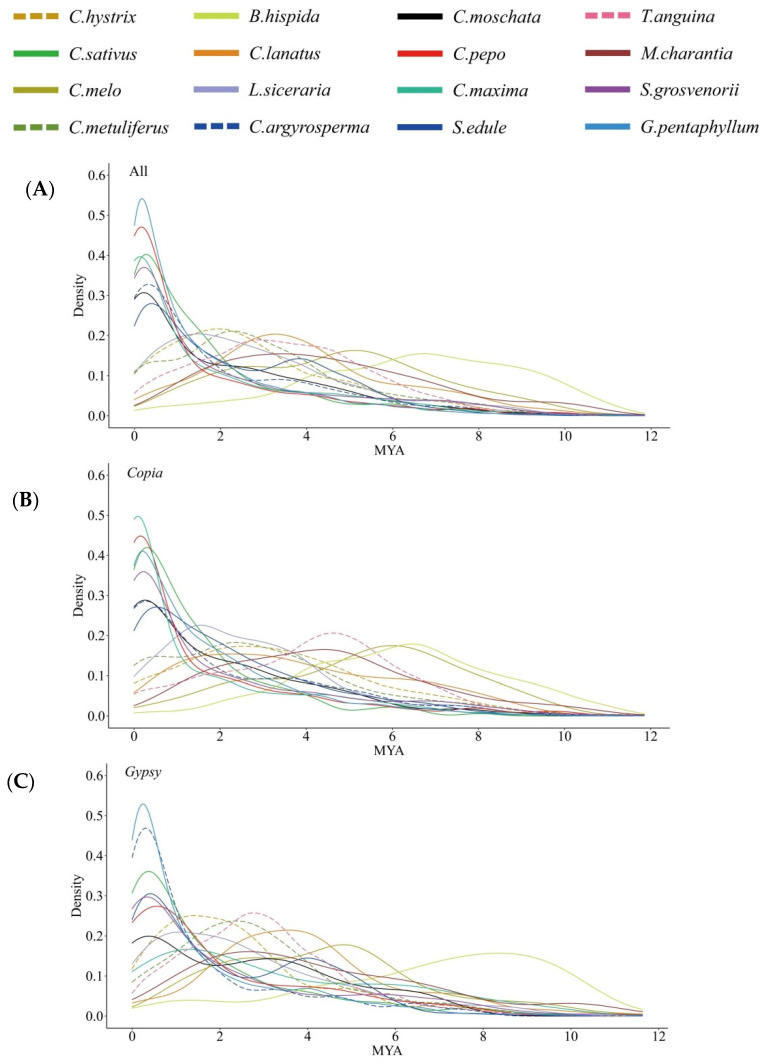
Distribution of full-length LTR-RTs in each species according to their estimated insertion ages (MY). (**A**) All full-length LTR-RTs; (**B**) *Copia* elements; (**C**) *Gypsy* elements.

Transposition time analysis of the full-length LTR-RTs presented that nearly all the identified elements inserted during the last 12 million years (MY) (Figure 4A). Detailed analysis of the amplification time of each species showed that the majority of the elements inserted after the speciation event of these species. At least one round of an LTR-RT burst occurred within each genome, with more rounds occurring in some species, such as the two rounds of bursts in *C. melo* and *S. edule*. The time of the LTR-RT burst varied dramatically among different species. Seven species showed recent expansions within the recent 0.5 MY, including *G. pentaphyllum*, *C. sativus*, *S. grosvenorii*, and the four *Cucurbita* species (*C. pepo*, *C. maxima*, *C. argyrosperma*, and *C. moschata*). The two largest genomes, *T. anguina* and *B. hispida*, showed very complex LTR-RT amplification patterns. The two latter both showed a long period of amplification events, with more ancient and fewer recent LTR-RT insertions in *B. hispida* (mean insertion age of 6.44 MY) than in *T. anguina* (mean insertion age of 3.55 MY) (Figure 4A). In most species, the insertion times of *Copia* and *Gypsy* elements showed large differences. Some species showed younger *Copia* insertion and older *Gypsy* insertion, such as in *B. hispida* and *C. maxima*. However, in other species, such as *T. anguina* and *C. melo*, the insertion time of *Gypsy* elements was more recent than that of *Copia* elements (Figure 4B,C). 

**Figure 5:**
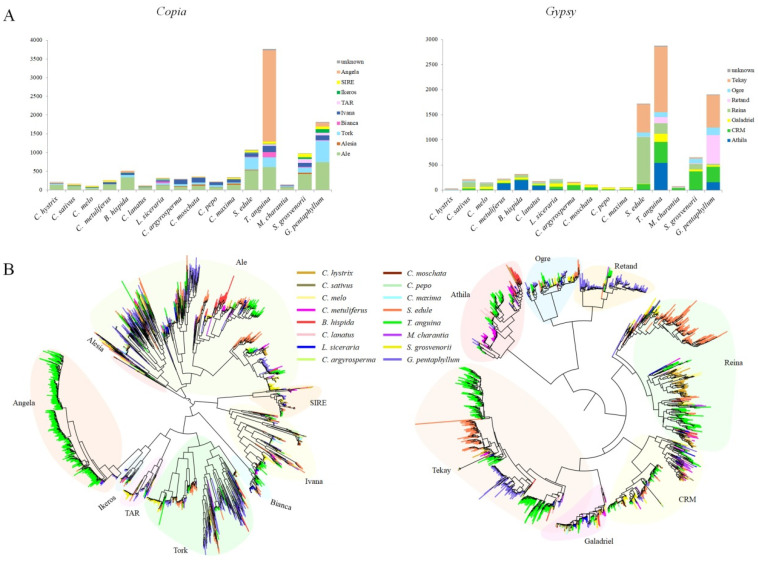
Diversity and evolution of LTR-RT lineages. (**A**) Number of full-length LTR-RTs belonging to different lineages of *Copia* and *Gypsy* superfamilies identified in the Cucurbitaceae genomes; (**B**) phylogenetic trees constructed based on reverse transcriptase domain sequences.

These full-length LTR-RTs were further classified as different lineages according to their RT protein domains to investigate the LTR-RTs in detail. The *Copia* and *Gypsy* elements were subclassified into seven and six lineages, respectively (Figure 5A). In general, among *Copia* elements, Ale, Angela, and Tork lineages were most predominant. The other four lineages were less abundant. However, remarkable variation in the LTR-RT composition of the lineages was observed within different species. For example, the Ale lineage accounted for 16.2% in *T. anguina* to 65.4% in *C. hystrix* of the full-length *Copia* elements. In *T. anguina*, whose genome had the largest number of LTR-RTs, Angela elements were most abundant, representing 64.8% of the *Copia* elements (Figure 5A). Analysis of *Gypsy* elements also showed large variation of lineage abundance in different species. In *T. anguina*, Tekay elements were most common, followed by CRM and Reina. In its close relative *S. edule*, Reina outnumbered Tekay, accounting for 54.0% of the *Gypsy* elements (Figure 5A).

**Figure 6:**
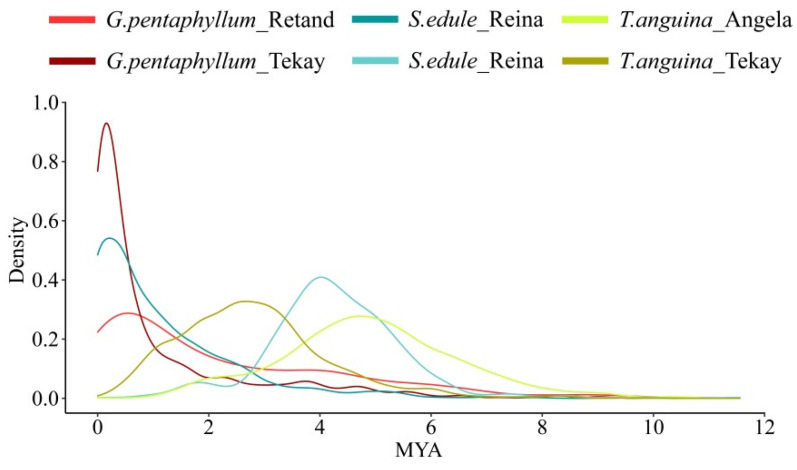
Insertion age of the amplified LTR-RT lineages in three species.

Two phylogenetic trees were developed according to their reverse transcriptase sequences to investigate the historical dynamics of these diverse lineages of *Copia* and *Gypsy* members in Cucurbitaceae genomes (Figure 5B). The trees were rooted with midpoint; thus, the elements represented by the sequences with farthest distance from the root were either the youngest elements or oldest ones. As shown in the evolutionary dendrograms, distinct patterns among different LTR-RT lineages were observed in each species. A number of species-specific bursts occurred for several lineages in different species. Combined with the insertion time of LTR-RTs in each species, the results suggested that the Angela and Tekay lineages were amplified relatively ancient in *T. anguina*, whereas a recent activity burst of Reina and another more ancient round of Tekay activity burst were examined in *S. edule*. In addition, Tekay and Retand lineages belonging to *Gypsy* superfamily underwent a recent burst in *G. pentaphyllum* (Figure 5B and Figure 6).

### 2.5. Impact of LTR-RTs on Gene Structure and Expression

**Figure 7:**
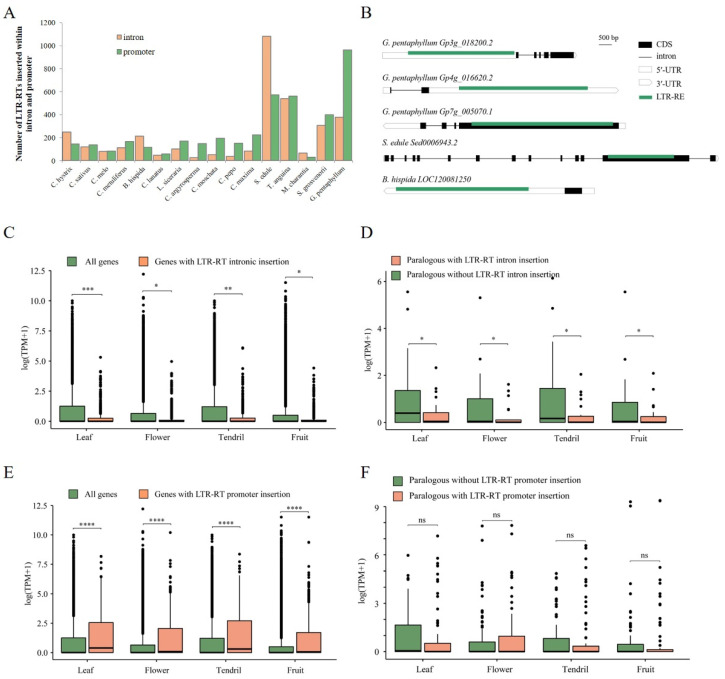
Impact of LTR-RTs on gene structure and expression. (**A**) Number of genes with LTR-RT insertions in introns or promoters; (**B**) LTR-RTs reside within the exons of five genes from three species; (**C**) comparison of gene expression levels between the genes with intronic LTR-RT insertions and the whole gene set in four tissues of *G. pentaphyllum*; (**D**) gene expression levels of paralogous gene pairs with or without intronic LTR-RT insertion in four tissues of *G. pentaphyllum*; (**E**) comparison of gene expression levels between the genes with promoter LTR-RT insertions and the whole gene set; (**F**) gene expression levels of paralogous gene pairs with or without promoter LTR-RT insertion. *p* **** < 0.0001; *p* *** < 0.001; *p* ** < 0.01; *p* * < 0.05. ns represents *p* > 0.05.

LTR-RTs located in the upstream region (promoter) and within the genes were analyzed to investigate the effects of LTR-RT retrotransposition on gene structure and function. A total of 26–1082 genes with intronic LTR-RTs insertions were detected in different species, whereas 31–965 genes were detected to have promoter LTR-RT insertions within these species (Figure 7A). It is interesting that five genes from three species had LTR-RTs inserted into their exons (Figure 7B). Among them, three genes had *Copia* insertions in *G. pentaphyllum*, whereas the other two had *Gypsy* insertions, one each in *B. hispida* and *S. edule*. Thus, LTR-RTs can be recruited as exons of functional genes in Cucurbitaceae species. 

The genes of *G. pentaphyllum* were analyzed in detail to evaluate the influence of LTR-RT insertion on gene expression. The average expression level of genes with intronic insertions was significantly lower than that of the total gene set in all the four examined tissues (*p* < 0.001, *p* < 0.01, or *p* < 0.05; paired *t*-test) (Figure 7C). In addition, the paralogous genes with their introns inserted of LTR-RTs showed significantly lower expression levels than their paralogs without intronic LTR-RT insertions (*p* < 0.05; paired *t*-test) (Figure 7D). The results indicated that the LTR-RTs inserted within genes were clear regulators for suppressing the gene expression.

The expression levels of genes with LTR-RTs inserted within their promoters were also compared with those of the whole gene set or the paralogous genes without promoter LTR-RT insertion. Surprisingly, the gene expression levels of promoter LTR-RT insertion were much significantly higher than those of the whole gene set (*p* < 0.001; paired *t*-test) (Figure 7E). However, paralogous gene pairs analysis showed no significant difference between the expression levels of genes with or without promoter LTR-RT insertion (*p* > 0.05). Detailed analysis revealed that this finding was because some genes with promoter LTR-RT insertion were downregulated compared with their paralogs having no insertion, whereas other genes with promoter LTR-RT insertion were upregulated compared with their paralogs having no insertion. Thus, this dual-directional regulation neutralized the difference in gene expression. For example, the gene *Gp11g_006840.1* had a *Gypsy* insertion within 4784 bp of the upstream region, whereas its paralog *Gp3g_016150.1* did not have this insertion. The expression level of *Gp11g_006840.1* was much lower than that of *Gp3g_016150.1* (Figure S2). By contrast, between another paralogous gene pair *Gp11g_003310.1* and *Gp11g_018200.2*, *Gp11g_003310.1* with promoter *Copia* insertion showed elevated gene expression (Figure S3). These results indicated that the LTR-RTs within the promoter region can suppress or enhance the gene expression, with the main function of enhancing gene expression. 

**Figure 8:**
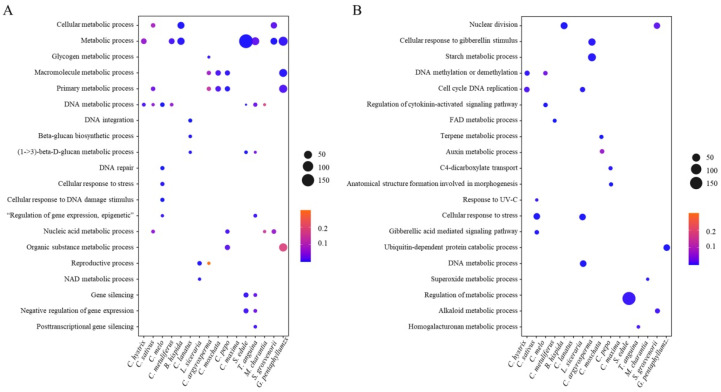
GO enrichment of genes associated with LTR-RT insertion. (**A**) Function enrichment of genes with TE insertion in introns. (**B**) Function enrichment of genes with TE insertions in promoters.

GO enrichment analysis for genes with intronic or promoter insertion of LTR-RTs was performed to understand the preference of the LTR-RT-associated gene function in Cucurbitaceae species. The results indicated that the LTR-RT-associated genes showed various functions, such as metabolism, response to stress, gene regulation, and DNA repair. The genes with intronic LTR-RT insertions tended to be related to metabolism (Figure 8A), whereas the genes with promoter LTR-RT insertions showed different functions among different species (Figure 8B).

## 3. Discussion

Plant genomes usually accumulate large amounts of LTR-RTs. Their diversity and the inherent propensity of their proliferation greatly contribute to the variation of plant genome size, structure, and function [35]. A number of Cucurbitaceae genomes have been sequenced and assembled, due to their great economic value as vegetable, fruit, or ornamental plants. These genomes allow us to compare the LTR-RT fractions among different species. This study focused on LTR-RT dynamics among the genomes of 16 species of the Cucurbitaceae family that differ more than fourfold in genome size. We demonstrated that LTR-RTs are also an essential source of genetic variation in Cucurbitaceae species. In plants, the changes in genome size of close related species can result from either polyploidization or TE amplification [36]. Large-scale transcriptome data show that the Cucurbitaceae species underwent four rounds of whole genome duplications before the last 10 MY [37]. Thus, recent polyploidy is unlikely a potential contributor for the observed variations in genome size. As expected, the LTR-RT component contributed significantly to the genome size variation of Cucurbitaceae species. The LTR-RT fraction was significantly positively correlated with the genome size. The two species with the largest genome size, *B. hispida* and *T. anguina*, had the most abundant LTR-RTs, accounting for 59.6% and 57.1% of their genome, respectively. 

However, further detailed analysis revealed a difference in the LTR-RT evolution between these two species. In *T. anguina*, the LTR-RT component and the full-length LTR-RTs were both abundant, whereas, although the LTR-RT fractions predominated in *B. hispida*, the full-length LTR-RTs were few. The number of full-length LTR-RTs in *B. hispida* was 914, which was only one-eighth of the number of full-length LTR-RTs in *T. anguina* (7833). Insertion age analysis showed that the LTR-RTs were inserted more ancient in these two species than in most of the other species. Specifically, the LTR-RTs in *B. hispida* were older than the majority of the LTR-RTs in *T. anguina*. Thus, although the full-length LTR-RTs were few, ancient LTR-RT bursts possibly occurred within the *B. hispida* genome. The active LTR-RTs usually harbor all the elements that facilitate retrotransposition upon transposition. Over evolutionary time, these LTR-RTs usually experienced a rapid evolutionary process, including truncations, nested insertions, mutations, and fragmentations [38]. These variations result in them not being identified as full-length LTR-RTs with loss of activity. 

The majority of the full-length LTR-RTs accumulated after the speciation event of all the studied species, implying very recent and possibly ongoing LTR-RT amplifications. Such recent LTR-RT proliferations were also reported in other species, such as rice, Brassica species, and spinach [39,40,41]. The recent RT amplification events possibly play important roles in the genome structure and evolution of these Cucurbitaceae species. In many cases, one or several TE types are greatly proliferated. For instance, five families of LTR-RTs represent about 80% of the maize RE dataset [42,43], and expansion of a specific CR1 retrotransposon family is associated with genome size increase and radiation in *Hydra* [44]. We also found that several species-specific lineages were also amplified at different times in the large genomes. For instance, the Angela and Tekay lineages with a great evolutionary age were amplified in *T. anguina*, whereas a recent activity burst of Reina and another more ancient round of Tekay activity burst were examined in *S. edule*. In addition, recent bursts of two *Gypsy* lineages, Tekay and Retand, occurred in *G. pentaphyllum*. The exact mechanism underlying the proliferation of certain RE families or lineages has not been elucidated, and it is widely believed that the suppression of these families or lineages reduced within the host genome probably because of external and/or internal stimuli [45,46]. These findings demonstrated that different genomes showed distinct LTR-RT evolutionary patterns, which were mainly due to different evolutionary processes undergone by each plant genome [47]. 

In addition to their influence on genome structure and evolution, increasing evidence shows that LTR-RTs can have tremendous impacts on gene structure and function. TEs inserted into exon or CDS usually lead to gene function loss. However, in the present study, we found the exons of five genes within three species harboring full-length LTR-RTs, suggesting that LTR-RTs can remodel genes by offering novel exons in these Cucurbitaceae species. This phenomenon is rare but has been reported in a number of animal and plant species. For example, a number of exons originated from TEs in human and mouse [19]. Thus, TEs, including LTR-RTs, provide novel materials to create new genes.

Except for recruiting as exons, LTR-RTs can also be associated with the regulatory elements of genes. Some LTR-RTs are inserted into introns, whereas others can reside in the upstream promoter regions of genes. Comparative transcriptome analysis showed that the expression levels of genes with intronic LTR-RT insertions were significantly lower than those of the whole gene set. Further analysis of paralogous gene pairs also confirmed the suppression function of LTR-RTs residing in the introns. The result is in accordance with a previous study on the tea genome, which showed that the paralogous genes with TE intronic insertions exhibited significantly lower expression levels than their paralogs without intronic TE insertions [3]. However, the expression levels of genes with promoter LTR-RT insertions exhibited opposite profiles. The genes with promoter LTR-RT insertions presented much higher expression levels compared with the whole genome set, whereas the paralogous gene pairs with or without LTR-RT insertion did not show a significant difference in expression levels. These results clearly reveal that the influence of LTR-RTs on gene expression depends on their location. The LTR-RTs within the introns can suppress gene expression, but LTR-RTs within the promoters show a complex influence on the adjacent genes, with the main function of enhancing gene expression. Previous reports demonstrated that LTR-RTs can promote the expression of adjacent genes [48,49]. TEs can function as promoters or other regulatory elements to alter gene expression and cause phenotypic variation in plants. In the present study, we found a number of full-length LTR-RTs within the upstream of genes that regulate gene expression and potentially alter plant phenotype. 

## 4. Materials and Methods

### 4.1. Plant Genome Sequences and Datasets

A total of 16 cucurbit species with available information on nuclear genomic sequences and gene annotation were used for annotation of LTR-RTs (Table S2). The genome assembly of *Begonia fuchsioides* from Begoniaceae was used as an outgroup for phylogenetic analysis of these species (Table S2), since the family Begoniaceae is the close relative of curcurbitaceae [50]. If multiple genome assembly versions were available for one species, we selected the assemblies with higher quality for analysis. We synthetically considered the contig N50, sequence coverage, and Benchmarking Universal Single-Copy Orthologous value for evaluation of the quality of the genome assemblies. 

### 4.2. Phylogenetic Analysis and Divergence Time Evaluation of These Species

In order to reconstruct the phylogenetic tree and evaluate the divergence time of the analyzed species, gene families were clustered using OrthoFinder (v1.1.5) [51]. A total of 497 single-copy orthologous genes were shared by the analyzed genomes. These protein sequences were connected successively and aligned using MAFFT [52]. Then, a phylogenetic tree was constructed using IQ_TREE [53] with *B. fuchsioides* as the root and then viewed in Figtree. The divergence time was predicted using MCMCTREE in the PAML package [54]. 

### 4.3. Identification of Full-Length LTR-RTs and Genome Annotation of LTR-RTs

Full-length LTR-RTs were initially predicted using LTR-FINDER [55] with the parameters “-D 20,000 -d 1000 -L 7000 -l 100 -p 20 -C -M 0.9” and the LTRharvest [56] program with the same settings. Using these parameters, the following putative LTR-RTs were identified: LTR size of 100–7000 bp, minimum and maximum distances between the two LTRs of 1000 and 20,000 bp, respectively, minimum length of exact match pair of 20, and similarity of the LTRs of 90%. The putative LTR-RTs were then imported to LTR_retriever [57] for further filtering, non-TGCA LTR-RT recovery, and annotation. On the basis of the LTR-retriever results, the identified intact LTR-RTs were first classified into the *Copia* and *Gypsy* superfamilies. Then, the protein domains of the elements belonging to different lineages of *Gypsy* or *Copia* superfamilies were analyzed using REXdb [9], which was implemented in the RepeatExplorer web server [58]. The identified full-length LTR-RTs with classification information were then utilized as a custom library to analyze the LTR-RT content of each genome using RepeatMasker. 

### 4.4. Insertion Time Estimation of Full-Length LTR-RTs

The insertion time of the intact LTR-RT was estimated using LTR_retriever on the basis of the nucleotide distance of 5′- and 3′-LTRs of each detected full-length LTR-RT. An average substitution rate of 4.5 × 10^−9^ substitutions per synonymous site per year from *B. hispida* was used to measure the insertion time [31].

### 4.5. Phylogenetic Analysis of LTR-RTs

The RT protein domain sequences of diverse lineages of the *Copia* and *Gypsy* superfamilies were collected, and redundant sequences were removed using CD-hit [59] with parameters “-c 1 -aL 0.9 -AL 10 -aS 1 -AS 1 -d 0”. Then, multiple sequence alignments were carried out using muscle [60] and used to generate phylogenetic trees by FastTree [61]. The trees were drawn and further edited using the iTOL online tool [62].

### 4.6. Analysis of Full-length LTR-RTs Associated with Genes

To investigate the relationship between genes and full-length LTR-RTs, we developed a python script to compare the position of the identified full-length LTR-RTs with the gene position in the GFF annotation file. We counted the number of LTR-RTs inserted into the exon, intron, and promoter (5′- terminal flanking 5000 bp of the genes) and extracted the corresponding gene ID. GO enrichment analysis of the genes with intronic or promoter insertion of LTR-RTs was carried out using the OmicShare tools (http://www.omicshare.com/tools, accessed on 15 December 2021).

Transcriptome data on the leaf, flower, tendril, and fruit of *Gynostemma pentaphyllum* were downloaded from the NCBI SRA with accession numbers SRR15100120, SRR15100121, SRR15100122, and SRR15111023, respectively, to evaluate the impact of LTR-RTs on the expression of adjacent genes. The gene expression level was quantified in TPM (transcripts per million). The expression levels of genes with LTR-RT insertions within their introns and promoters were compared with the expression levels of all genes in the four tissues. In addition, the expression levels of paralogous genes were compared with the expression of one gene harboring intronic/promoter LTR-RT insertion, with the other one having no insertion. The paralogous genes were detected using SynOrths software [63].

## 5. Conclusions

This study comprehensively characterized the LTR-RT landscape, evolution, and function in Cucurbitaceae species. Our data provide a holistic view of LTR-RTs and their unique roles in Cucurbitaceae species. Considerable variation was found in the compositions and abundance of LTR-RTs. Distinct patterns of evolutionary dynamics of different LTR-RT lineages were observed in each species. The recent LTR-RT amplification events reflect the ongoing rapid evolution of these genomes. The different influences of LTR-RTs on related genes, especially those having intronic and promoter insertions, were also demonstrated. Thus, LTR-RTs contributed significantly to genome structure, evolution, and gene regulation in Cucurbitaceae species. Cucurbitaceae species proved to be an attractive model system for studies of TE-driven genome expansion, considering the diversity in the accumulation and genomic distribution of LTR-RTs. This study may serve as a reference for further research on LTR-RTs influencing gene function and perhaps plant phenotypes. 

## Supplementary Materials

The following supporting information can be downloaded at: https://www.mdpi.com/article/10.3390/ijms231710158/s1.
