# Supplementary material for: Impact of LTR-Retrotransposons on Genome Structure, Evolution, and Function in Curcurbitaceae Species
Source: Int J Mol Sci. 2022 Sep 5;23(17):10158. doi: 10.3390/ijms231710158 (PMC9456015; doi:10.3390/ijms231710158)
Supplement: Supplementary file 1 [file ijms-23-10158-s001.zip › ijms-1877707-supplementary.pdf]

Table S1 Detection and characteristics of full-length LTR-RTs in Cucurbitaceae species

Species	Assembled genome size	Number of full-length LTR-RTs	Cumulative length	Number/Mb	Number of each type			Average length of each type (bp)		
					Copia	*Gypsy*	Unknown	*Copia*	*Gypsy*	Unknown
Cucumis hystrix	289	625	3,275,727	2.2	211	365	49	5520	5357	3175
Cucumis sativus	226.2	450	2,645,514	2.0	164	215	71	6104	6754	2625
Cucumis melo	417	269	1,494,679	0.6	108	148	13	6052	5436	2818
Cucumis metuliferus	316.8	522	3,810,409	1.6	261	233	28	5774	9225	5500
Benincasa hispida	913	914	6,101,374	1.0	514	319	81	5958	8289	4861
Citrullus lanatus	396.4	349	2,788,382	0.9	118	172	59	6272	9092	8211
Lagenaria siceraria	313.4	646	3,682,561	2.1	321	221	104	5749	6578	3689
Cucurbita argyrosperma	255.2	528	2,523,365	2.1	289	157	82	4631	5776	3393
Cucurbita moschata	269.9	605	2,786,293	2.2	358	116	131	4876	6167	2484
Cucurbita pepo	261.4	339	1,530,852	1.3	224	49	66	4928	5507	2380
Cucurbita maxima	271.4	472	2,300,796	1.7	333	49	90	5235	6498	2657
Sechium edule	606.4	3567	23,790,490	5.9	1069	1723	775	5531	7310	6800
Trichosanthes anguina	919.8	7833	63,807,282	8.5	3763	2877	1193	8579	8640	5590
Momordica charantia	285.6	242	1,230,197	0.8	136	74	32	4760	6117	4070
Siraitia grosvenorii	467.1	1836	10,585,418	3.9	976	654	206	5505	6745	3890
Gynostemma pentaphyllum	582.9	4739	34,541,089	8.1	1812	1908	1019	5597	9505	6141

Table S2 Detailed information of the genomes of 16 Cucurbitaceae species and 1 related Begoniaceae species

Species	Genus	Tribe	Family	Estimated genome size (Mb)	Assembled genome size (Mb)	Genome download linkage
Cucumis hystrix	*Cucumis*	Benincaseae	Cucurbitaceae	416.0	289.0	https://figshare.com/articles/dataset/Genome_assembly_of_Cucumis_hystrix/13377671
Cucumis sativus	*Cucumis*	Benincaseae	Cucurbitaceae	350.0	226.6	https://www.ncbi.nlm.nih.gov/genome/1639?genome_assembly_id=749658
Cucumis melo	*Cucumis*	Benincaseae	Cucurbitaceae	450.0	417.0	ftp://cucurbitgenomics.org/pub/cucurbit/genome/melon/v3.6.1/
Cucumis metuliferus	*Cucumis*	Benincaseae	Cucurbitaceae	368.0	316.8	https://ngdc.cncb.ac.cn/gwh/Assembly/10134/show
Benincasa hispida	*Benincasa*	Benincaseae	Cucurbitaceae	1030.0	913.0	https://www.ncbi.nlm.nih.gov/genome/18245?genome_assembly_id=747481
Citrullus lanatus	*Citrullus*	Benincaseae	Cucurbitaceae	419.2	396.4	ftp://cucurbitgenomics.org/pub/cucurbit/genome/watermelon/WCG/
Lagenaria siceraria	*Lagenaria*	Benincaseae	Cucurbitaceae	334.0	313.4	http://cucurbitgenomics.org/organism/13
Cucurbita argyrosperma	*Cucurbita*	Cucurbiteae	Cucurbitaceae	238.0	255.2	https://www.ncbi.nlm.nih.gov/genome/75408?genome_assembly_id=1634948
Cucurbita moschata	*Cucurbita*	Cucurbiteae	Cucurbitaceae	372.0	269.9	https://www.ncbi.nlm.nih.gov/genome/18321?genome_assembly_id=351850
Cucurbita pepo	*Cucurbita*	Cucurbiteae	Cucurbitaceae	283.0	261.4	https://www.ncbi.nlm.nih.gov/genome/12199?genome_assembly_id=380250

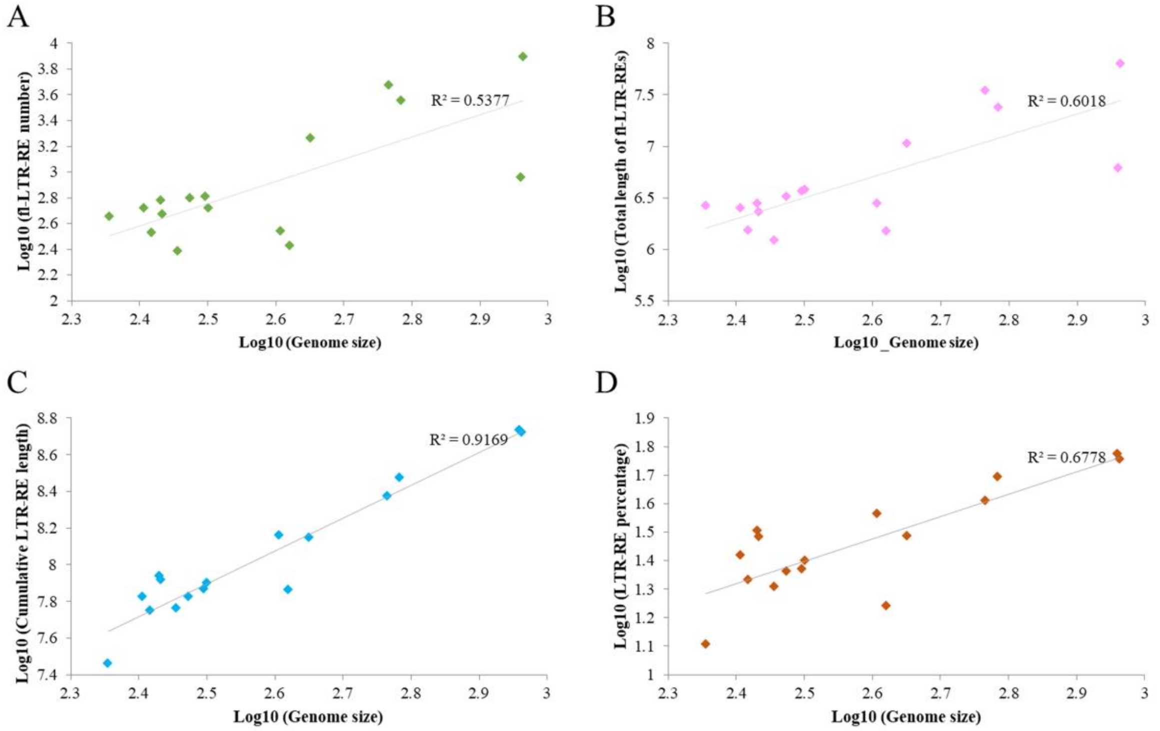

Figure S1. Correlation between genome size and full-length LTR-RT fraction or LTR-RT fractions in Cucurbitaceae species. (**A**) Genome size versus number of full-length LTR-RTs; (**B**) genome size versus cumulative length of full-length LTR-RTs; (**C**) genome size versus the total length of LTR-RTs including full-length and fractionated elements; (**D**) genome size versus the percentage of genomes occupied by LTR-RTs including full-length and fractionated elements.

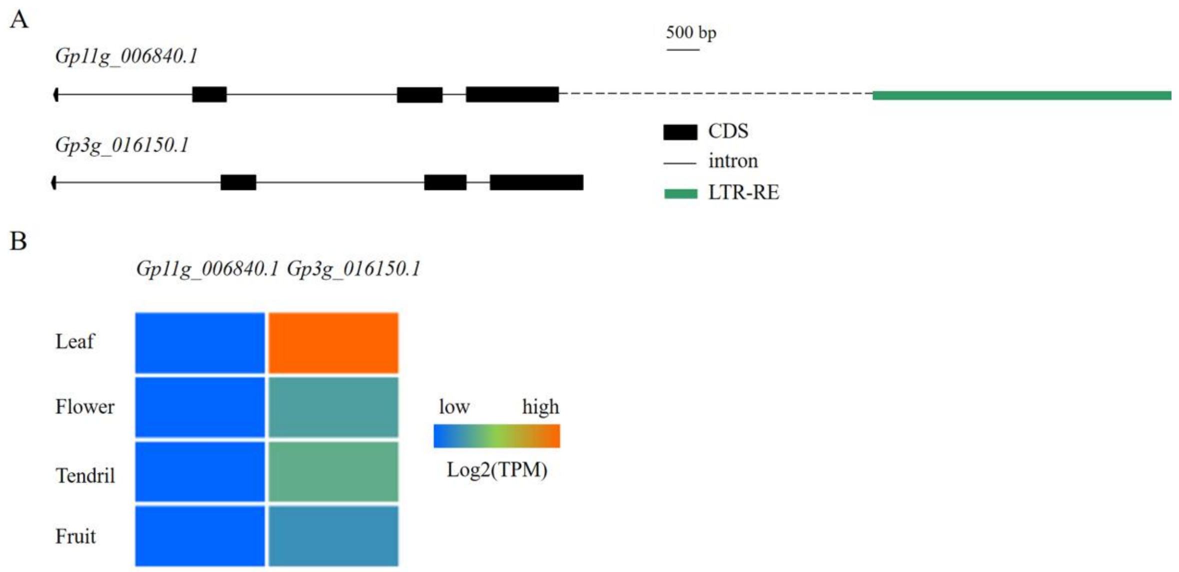

Figure S2. Example of gene with promoter LTR-RT insertion showed lower expression level than its paralog. (**A**) Gene structure of a paralogous gene pair with or without LTR-RT insertion; (**B**) Expression level of this paralogous gene pair.

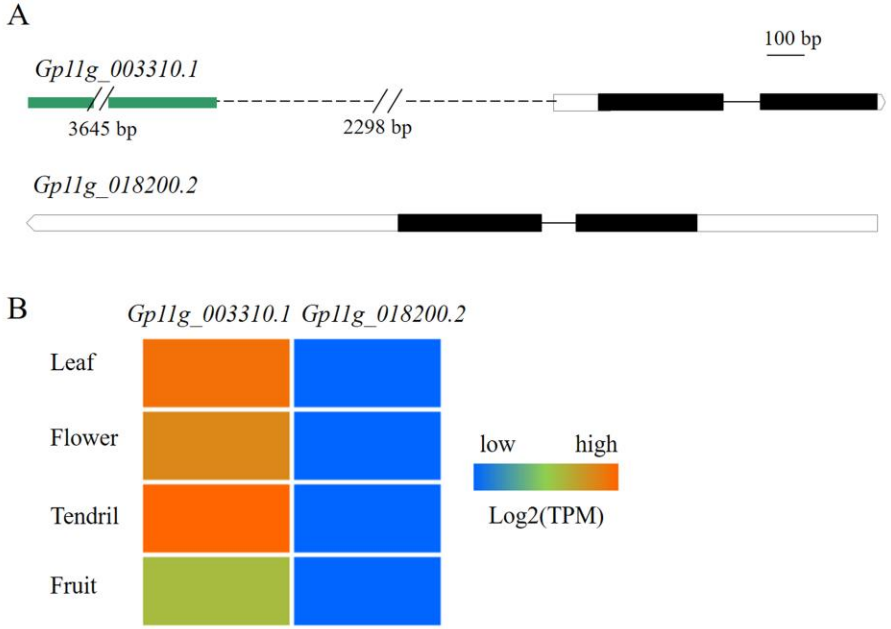

Figure S3. Example of gene with promoter LTR-RT insertion showed elevated expression level than its paralog. (**A**) Gene structure of a paralogous gene pair with or without LTR-RT insertion; (**B**) Expression level of this paralogous gene pair.
